# Conspecificity of two morphologically distinct calcified red algae from the northwest Pacific Ocean: *Galaxaura pacifica* and *G. filamentosa* (Galaxauraceae, Rhodophyta)

**DOI:** 10.1186/1999-3110-54-1

**Published:** 2013-07-18

**Authors:** Shao-Lun Liu, Lawrence M Liao, Wei-Lung Wang

**Affiliations:** 1 Department of Life Science, Tunghai University, Taichung, 407 Taiwan; 2 Graduate School of Biosphere Science, Hiroshima University, 1-4-4 Kagamiyama, Higashi-Hiroshima, 739-8528 Japan; 3 Department of Biology, National Changhua University of Education, Changhua, 500 Taiwan

**Keywords:** *G. filamentosa*, *G. pacifica*, Galaxauraceae, *rbc* L, Rhodophyta, Taiwan, The Philippines

## Abstract

**Background:**

Members of the calcified red algal genus, *Galaxaura*, are distributed predominantly in warm temperate, subtropical, and tropical regions worldwide. The capacity of these algae to form calcified thalli could play a critical role in the carbon cycle of these ecosystems. Previous studies have suggested that the reported species diversity of *Galaxaura* may be exaggerated due to a lack of knowledge regarding external morphological differences between gametophytic and tetrasporophytic plants (or among different life stages) of a single species.

**Results:**

To examine this issue, this study collected specimens of two morphologically distinct *Galaxaura* from Taiwan and the Philippines. These specimens were initially identified as two species (*G. pacifica* Tanaka and *G. filamentosa* Chou ex Taylor) based on their morphological features. Our molecular analyses, however, unexpectedly showed that these two specimens shared 100% identical *rbc* L sequences, indicating that they represented a single species comprising two distinct external morphologies. Furthermore, our extensive observations and molecular analyses on several specimens from different locations in southern Taiwan has revealed that these morphological differences could be due to seasonal variation.

**Conclusions:**

This study proposes that *G.* “*filamentosa*” from the Philippines could represent the remnants of the lower villous part of older gametophytic plants of *G. pacifica* after senescence of the upper smooth part of the thallus. As such we propose that these two previously distinct algal species from the northwest Pacific Ocean as a single species, *G. pacifica*. This study shows that the biodiversity of the calcified red algae *Galaxaura* could be overestimated without the assistance of molecular tools. Additionally, this study provides insights into the biodiversity and unique biology of the calcified red algae *Galaxaura*.

**Electronic supplementary material:**

The online version of this article (doi:10.1186/1999-3110-54-1) contains supplementary material, which is available to authorized users.

## Background

Species of the calcified red algal family Galaxauraceae are distributed widely throughout the shallow marine waters of the warm temperate, subtropical, and tropical regions (e.g., Littler and Littler, 1997;Abbott 1999;Huisman, 2006). The unique ability of these red algae to incorporate calcium carbonate into their thalli makes them critical elements in the carbon budget, biomineralization, reef building processes, and coastal marine ecosystems. Research related to their biodiversity is necessary to understand their contribution to the carbon cycle of tropical marine ecosystems because calcium carbonate deposition in algae is affected by environmental conditions and differs from one species to another (Stanley et al., 2010). Seven genera were historically recognized in the family Galaxauraceae, including *Actinotrichia* Decaisne, 1842, *Galaxaura* Lamouroux, 1812, *Tricleocarpa* Huisman and Borowitzka 1990, *Gloiophloea* J. Agardh, 1872, *Scinaia*,^1822^, *Nothogenia* Montagne, 1843, and *Whidbeyella* Setchell and Gardner 1903 (reviewed in Huisman, J.M 2006). Huisman et al*.* (2004a) used molecular analyses to resurrect the genus *Dichotomaria* Lamarck 1816 from synonymy under *Galaxaura*, and split the family Galaxauraceae into two different families, Galaxauraceae and Scinaiaceae. The former currently comprises four calcified genera, *Actinotrichia*, *Dichotomaria*, *Galaxaura*, and *Tricleocarpa*; whereas the latter consists of four non-calcified genera, *Gloiophloea*, *Scinaia*, *Nothogenia*, and *Whidbeyella*.

The genus *Galaxaura* was first erected by Lamouroux (1812). Kjellman (1900) recognized 62 species and described 47 of these species as new, suggesting that the genus *Galaxaura* is extremely diverse. However, later studies challenged this taxonomic system. Howe (1917,1918) suggested that the species diversity of *Galaxaura* could be inflated because of the different external morphologies in the sexual (i.e., gametophytic) and sporophytic (i.e., tetrasporophytic) stages, referred to as the dimorphic life history (e.g., Wang et al. 2005). Guided by this concept, two fundamental studies that investigated the calcified red algal family Galaxauraceae in the Indian Ocean and Australian region reduced a significant number of different species to a few pantropical species such as *Galaxaura rugosa*, *G. marginata* (= *Dichotomaria marginata*), and *G. obtusata* (= *D. obtusata* ) (Papenfuss et al. 1982;Huisman and Borowitzka 1990). The elucidation of a possible relationship between two morphologically different (e.g., gametophytic vs. tetrasporophytic) species of the calcified red algal genus *Galaxaura* remains largely unexplored, although the concept of the dimorphic life history was only recently proven and the synonymy of some *Galaxaura* species has been demonstrated (Huisman et al. 2004b;Kurihara et al. 2005;Wang et al. 2005). Wang et al. (2005) suggested that the genus *Galaxaura* should have more diverse species in the tropical oceans and that the synonymy of numerous species based solely on morphological observations should be reassessed carefully after showing that the monophyly of a pan-tropical species, *G. rugosa*, was not supported by their molecular analyses.

To examine this issue, we collected two specimens of the genus *Galaxaura* from Taiwan and the Philippines. Based on their morphological characters, the specimens were initially identified as two different species. They were identified according to the taxonomic keys in the literature as *Galaxaura pacifica* Tanaka (Tanaka, 1935) and *G. filamentosa* Chou ex Taylor (Chou, 1945). The thallus of *G. pacifica* comprises a lower villous portion where branches are very hairy (i.e., villous) and composed of numerous assimilatory filaments and an upper smooth portion where branches are very smooth (i.e., glabrous) and lack assimilatory filaments (Tanaka, 1935,1936). In contrast, the branches of *G. filamentosa* are extremely villous throughout the thallus (Chou, 1945). Thus, *G. pacifica* was considered to be in the sexual phase (i.e., gametophytic plants) (Tanaka 1935), whereas *G. filamentosa* was speculated to be in the asexual phase (i.e., tetrasporophytic plants) (Chou, 1945), but in separate species because of their distinct morphologies. These two species have been previously documented to occur in the coral reef areas of Taiwan by Tanaka (1935) and Itono (1977). This study examined these specimens morphologically and compared their *rbc* L sequences, and additionally examined morphological variation in several samples from different locations in southern Taiwan. The results of these studies are presented herein.

## Methods

### Sample collection and morphological observations

Collections were made by snorkeling or SCUBA diving. Samples for morphological study were preserved in 5-10% formalin-seawater or pressed on herbarium sheets while materials used in the molecular study were preserved in 95% alcohol or desiccated in silica gel powder. Examined specimens were deposited at the herbarium of Department of Life Science, Tunghai University (TU), Taiwan. For morphological observations, materials were first decalcified in 1% HCl solution, then either squashed or sectioned by hand or a Leica CM1850 freezer microtome. When using the freezer microtome, the thickness of sections was set to 20-40 μm. Sections were stained with 1% aniline blue acidified with 1% HCl and mounted in 25-30% Karo® syrup (Englewood Cliffs, USA), or treated with aceto-iron-hematoxylin-chloral hydrate and mounted in 50% Hoyer’s mounting medium following the descriptions in Wang et al. (2005). Photomicrographs of specimens were taken on a Pixera Penguin 600CL digital camera (Tokyo, Japan) and a Nikon 995 digital camera (Tokyo, Japan).

### Nucleic acid extraction, polymerase chain reaction, and sequencing

DNA was extracted using the DNeasy Plant Mini Kit (Qiagen, Valencia, CA, USA) following the instructions of the manufacturer. PCR and sequencing followed the procedure described in Wang et al. (2005). Two newly obtained *rbc* L sequences were directly submitted to NCBI as GenBank accession numbers JQ814750 and JQ814751. Additional *rbc* L sequences were retrieved from GenBank, aligned with the software MUSCLE (Edgar, 2004), and then exported into the software Garli (Zwickl 2006) for phylogenetic analyses. The *rbc* L sequences available from GenBank and used in this study are listed in Table 1. A total of 45 different *rbc* L sequences of red algal species in the family Galaxauraceae around the world was selected for our phylogenetic analysis, together with two additional taxa belonging to the family Scinaiaceae as the outgroup (Table 1).

**Table 1:** **List of 47 different** ***rbc*** **L sequences used in the maximum likelihood phylogenetic analysis, their collection information and source, and accession numbers in GenBank**

Species	Collection information and source	Accession number
Galaxauraceae		
*A. fragilis* (Forsskål) Børgesen	Wanlitung, Kenting National Park (KNP), S. Taiwan (Wang et al. 2005)	AY688009
*A. fragilis* (Forsskål) Børgesen	Sulpa Island, Cebu, Philippines (Wang et al. 2005)	AY688010
*A. fragilis* (Forsskål) Børgesen	Bandar Khayran (ssMUS-003), Captial Area, Oman (Liu and Wang, 2009)	EU095253
*A. robusta* Itono	Outlet of the 3^rd^ Nuclear Power Plant, KNP, S. Taiwan (Wang et al., 2005)	AY688011
*A. taiwanica* Liu et Wang	Chiupeng, KNP, Taiwan (Liu and Wang, 2009)	EU105470
*D. apiculata* Kjellman	Shika, Hakui, Ishikawa Prefecture, Japan (Kurihara et al., 2005)	AB117627
*D. australis* (Sond.) Huisman, Harper et Saunders	Jervis Bay, N.S.W., Australia (Kurihara and Huisman, 2006)	AB258440
*D. australis* (Sond.) Huisman, Harper et Saunders	Shark Pt., Sydney, N.S.W., Australia (Kurihara and Huisman, 2006)	AB258442
*D. australis* (Sond.) Huisman, Harper et Saunders	Sorrento Back Beach, Victoria, Australia (Kurihara and Huisman, 2006)	AB258444
*D. diesingiana (Zanardini) Huisman, Harper et Saunders*	Sharks Bay, Port Alfred, Cape Province, South Africa (Wang et al., 2005)	AY688026
*D. falcata Kjellman*	Toji, Shimoda, Shizouka Prefecture, Japan (Kurihara et al., 2005)	AB117629
*D. marginata* (Ellis et Solander) Lamarck	Malang Lagoon, Papua New Guinea (Wang et al., 2005)	AY688018
*D. marginata* (Ellis et Solander) Lamarck	Ookataura, Hachijo Island, Japan (Kurihara et al., 2005)	AB117630
*D.* “*latifolia*” Tanaka	Dahsianglan, N.E. Taiwan (Wang et al., 2005)	AY688021
*D. marginata* (Ellis et Solander) Lamarck	Sulpa Island, Cebu, Philippines (Wang et al., 2005)	AY688017
*D. marginata* (Ellis et Solander) Lamarck	Anorde Rocks, St. prorjono, Guadeloupe (Wang et al., 2005)	AY688019
*D.* “*elegans*” Tanaka	Sail Rock, KNP, S. Taiwan (Wang et al., 2005)	AY688020
*D.* “*veprecula*” Kjellman	Puerto Libertad, Sonora, Gulf of California, Mexico (Wang et al., 2005)	AY688022
*D. obtusata* (Ellis et Solander) Lamarck	Palm Beach, Natal, South Africa (Wang et al., 2005)	AY688025
*D. obtusata* (Ellis et Solander) Lamarck	Itoman, Okinawa Island, Okinawa Prefecture, Japan (Kurihara et al., 2005)	AB117632
*D. obtusata* (Ellis et Solander) Lamarck	Parakeet Bay, Rottnest Island, W.A., Australia (Kurihara and Huisman, 2006)	AB258447
*D. obtusata* (Ellis et Solander) Lamarck	Tiaoshih, KNP, S. Taiwan (Wang et al., 2005)	AY688024
*D. papillata* Tanaka	Reihoku, Amakusa, Kumamoto Prefecture, Japan (Kurihara et al., 2005)	AB117631
*D. spathulata* (Kjellman) Kurihara et Huisman	Green Island, Rotnnest Island, W.A., Australia (Kurihara and Huisman, 2006)	AB258446
*D. tenera* (Kjellman) Huisman, Harper et Saunders	Palm Beach, Natal, South Africa (Wang et al., 2005)	AY688023
*G. divaricata* (Linnaeus) Huisman et Townsend	Miyano-hama, Chichi-jima, Bonin Islands, Tokyo, Japan (Kurihara et al., 2005)	AB117628
*G. divaricata* (Linnaeus) Huisman et Townsend	Dabaisa, Green Island, E. Taiwan (Wang et al., 2005)	AY688007
*G. cuculligera* Kjellman	Reihoku, Amakusa, Kumamoto Prefecture, Japan (Kurihara et al., 2005)	AB117633
*G.* “*filamentosa*” Chou ex Taylor	Bulusan, Sorsogon, Phillipines (Wang et al., 2005)	AY688004
*G.* “*filamentosa*” Chou ex Taylor	Five Caves, Orchid Island, S. Taiwan (Wang et al., 2005)	AY688006
*G. pacifica* Tanaka	Higashi Port, Haha-jima, Bonin Islands, Tokyo (Kurihara et al., 2005)	AB117638
*G. pacifica* Tanaka	Wukeuitung, Xiao-Liu-Qiu Island, S. Taiwan (Wang et al., 2005)	AY688005
*G. pacifica* Tanaka	Small Port, KNP, S. Taiwan; coll. S.-L. Liu and C.-S. Lin, 13.iii.2003 (This study)	JQ814751
*G. pacifica* Tanaka	Sail Rock, KNP, S. Taiwan; coll. S.-L. Liu and W.-L. Wang, 26.ii.2012 (This study)	JQ814750
*G. rugosa* (Ellis et Solander) Lamourou*x*	Green Island, W.A., Australia (Kurihara and Huisman, 2006)	AB258448
*G. rugosa* (Ellis et Solander) Lamouroux	Chiupeng, KNP, S. Taiwan (Wang et al., 2005)	AY687999
*G. rugosa* (Ellis et Solander) Lamouroux	Bulusan, Sorsogon, Philippines (Wang et al., 2005)	AY688000
*G. rugosa* (Ellis et Solander) Lamouroux	Content Key, Florida Keys, Florida Bay, Florida, USA (Wang et al., 2005)	AY688001
*G. rugosa* (Ellis et Solander) Lamouroux	St. Frorljors, Guadeloupe (Wang et al., 2005)	AY688002
*G. rugosa* (Ellis et Solander) Lamouroux	Las Playas Piedras, Bahia San Carlos, N of Guyamas, Sonora, Gulf of California, Mexico (Wang et al., 2005)	AY688003
*T. cylindrica* (Ellis et Solander) Huisman et Borowitzka	Guadeloupe (Wang et al., 2005)	AY688012
*T. cylindrica* (Ellis et Solander) Huisman et Borowitzka	Dabaisha, Green Island, E. Taiwan (Wang et al., 2005)	AY688013
*T. cylindrica* (Ellis et Solander) Huisman et Borowitzka	Penlung Bridge, N.E. Taiwan (Wang et al., 2005)	AY688014
*T. cylindrica* (Ellis et Solander) Huisman et Borowitzka	Sonora, Gulf of California, Mexico (Wang et al., 2005)	AY688015
*T. fragilis* (Linnaeus) Huisman et Townsend	Wanlitung, KNP, S. Taiwan (Wang et al., 2005)	AY688016
**Scinaiaceae**		
*Scinaia okamurae* (Setchell) Huisman	Omaezaki, Shizouka Prefecture, Japan (Kurihara et al., 2005)	AB258450
*Scinaia latifrons* Howe	Tsurumi, Ooiita Prefecture, Japan (Kurihara et al., 2005)	AB258449

### Phylogenetic analysis

Phylogenetic analyses and bootstrapping statistical analyses were performed with a maximum likelihood (ML) approach using the software Garli. The calculation of bootstrap proportion values was conducted as described in Felsenstein (1985). A total of 100 bootstrapping replicates was implemented with a ML approach. The general time reversible (GTR) nucleotide substitution model with gamma distribution was chosen to calibrate the nucleotide substitution rate. The six different nucleotide substitution rates and the proportion of invariant sites were empirically estimated from our data using the software Garli with default settings. Phylogenetic inference was also conducted with a Bayesian method using MrBayes v3.1.2 (Huelsenbeck and Ronquist, 2001). Briefly, two parallel runs were executed, and each run consisted of four chains (MCMC sampling; one hot and three cold) and one tree was sampled every 1,000 generations for 1,000,000 generations. In total, 1,000 samples (or trees) were obtained and 25% of samples were the “burn-in” of the chain. After 750,000 generations, trees were saved and 250 of them with standard deviation of split frequency < 0.01 were used later for the calculation of posterior probability. Uncorrected Proportional distance (uncorrected *P*-distance) method was used to calculate the nucleotide difference of *rbc* L sequences among the individuals of *G. pacifica*.

### Statistical analysis

To see if there is any difference in terms of ratio between the height of the glabrous part and the height of the villous part of plants among specimens from different locations, the pairwise *t*-test was applied using the statistical software R (http://www.r-project.org). The significant level was set to 0.05.

## Results

### Molecular analyses revealing that *G. pacifica* and *G. “filamentosa”* are the same species

**Figure 1:**
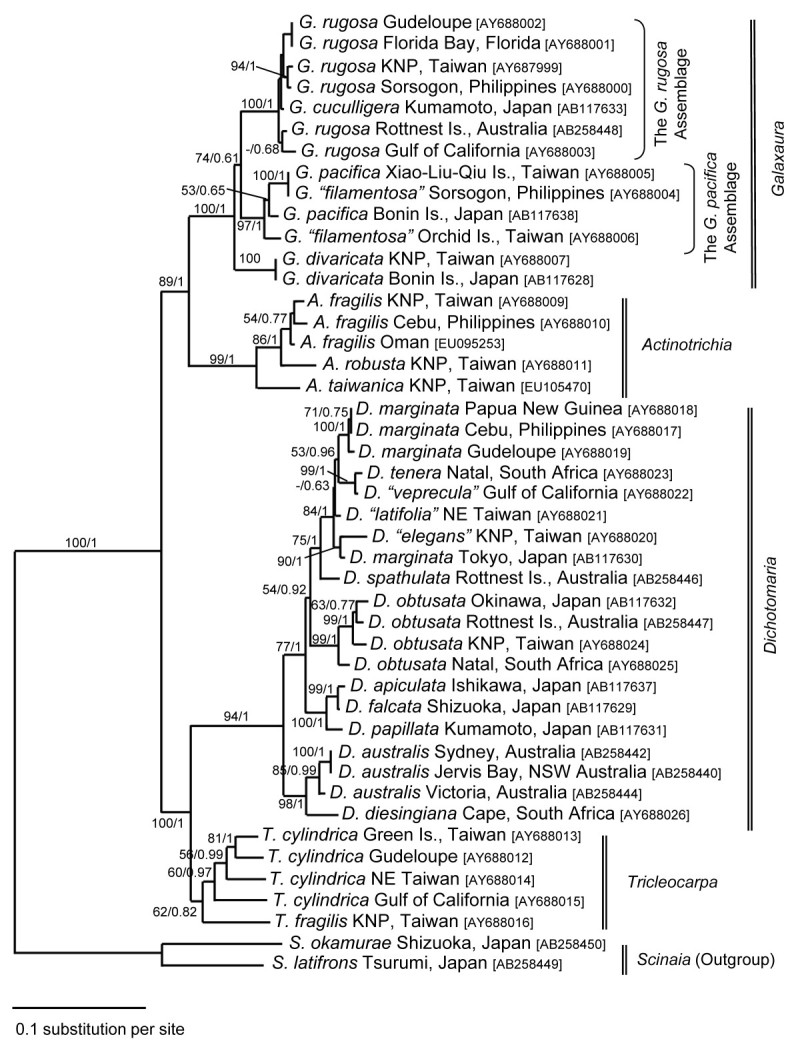
**Maximum-likelihood phylogenetic tree of the red algal family Galaxauraceae with two species of Scinaiaceae as outgroup.** Statistical supports are shown on branches. The first value is 100 replicates of bootstrapping proportion values (> 50%) using ML analyses. The second value is posterior probability (>0.5) using Bayesian analyses.

The *rbc* L sequences analyzed in this study were obtained primarily from GenBank, NCBI (Table 1). Two taxa of the Scinaiaceae were used as the outgroup. The analyzed matrix included 1407 characters, but some taxa only comprised partial characters because of their incomplete *rbc* L sequences. The topology of the ML tree was largely identical with that of the Bayesian tree, so only the ML tree is presented (Figure 1). Four major clades (representing the genera *Actinotrichia*, *Dichotomaria*, *Galaxaura*, and *Tricleocarpa*) with statistical support were identified by molecular analysis (Figure 1). For the intergeneric relationship, *Actinotrichia* and *Galaxaura* grouped together as a monophyletic group, referred as to the *Actinotrichia*/*Galaxaura* clade, whereas *Tricleocarpa* and *Dichotomaria* grouped together as the other monophyletic group, referred to as the *Tricleocarpa*/*Dichotomaria* clade. The *Actinotrichia*/*Galaxaura* clade is the sister lineage of the *Tricleocarpa*/*Dichotomaria* clade. The intergeneric relationship among these genera received high statistical support (Figure 1). Three distinct sub-clades were observed in our phylogenetic analyses in the genus *Galaxaura* (Figure 1). All of them received high statistical support (99%-100%). The basal clade of the genus *Galaxaura* is *G. divaricata* from Japan and Taiwan (Figure 1). The second clade of the genus *Galaxaura* consists of three distinct lineages that are related to the species *G. pacifica* obtained from its type locality in the Bonin Islands (Ogasawarajima), Japan, and are referred to as the *G. pacifica* assemblage (Figure 1). Within the *G. pacifica* assemblage, we unexpectedly discovered that two morphologically distinct species, *G. pacifica* from Xiao-Liu-Qiu Island, Taiwan and *G.* “*filamentosa*” from Sorsogon, the Philippines shared identical *rbc* L sequences, indicating that they are a single species. The *rbc* L sequences of the specimens from Xiao-Liu-Qiu Island (Taiwan) and Sorsogon (the Philippines) differ by approximately 2% (27 of 1380 nucleotides) from that of *G. pacifica* collected from the southernmost insular Japan (the type locality of *G. pacifica*). Another specimen that was morphologically identified as *G.* “*filamentosa*” occupies the basal lineage of the *G. pacifica* assemblage (Figure 1). The last clade of the genus *Galaxaura* comprises the generitype species, *G. rugosa*, from Guadeloupe, which is geographically close to its type locality in Jamaica, and other *Galaxaura rugosa*-related complexes from various places around the world, collectively referred to as the *G. rugosa* assemblage (Figure 1). Further morphological examinations are necessary for identification of different species because only *G. cuculligera* was recognized within the *G. rugosa* assemblage (Figure 1).

### *Rbc* L sequence analyses revealing seasonal variation of the external morphology in *G. pacifica*

**Figure 2:**
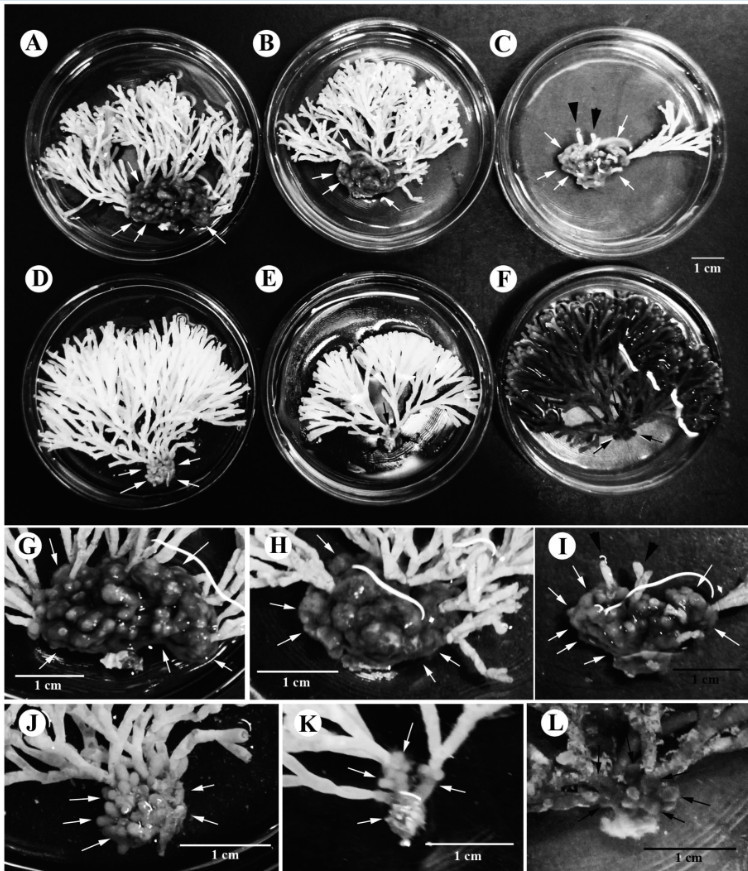
**Variations of the external morphologies of gametophytic** ***G. pacifica*** **for the specimens collected from three different locations in southern Taiwan.** **(A-C)** Specimens from Xiao-Liu-Qiu Island, southern Taiwan in summer showing different size of the villous basal portion (arrows) and different density of the glabrous upper portion. One specimen **(C)** even showed the decay of glabrous upper portion (arrowheads); **(D-E)** Specimens from Small Port, Kenting National Park (KNP), southern Taiwan in winter showing smaller villous basal portion (arrows) and highly branched glabrous upper portion of material from Wukeuitung, Xiao-Liu-Qiu Island, southern Taiwan in summer; **(F)** Specimen from Sail Rock, KNP, southern Taiwan in winter showing tiny villous basal portion (arrows) and highly branched glabrous upper portion. **(G-I)** Magnification of the villous basal portion (arrows) for the specimens from Wukeuitung, Xiao-Liu-Qiu Island, southern Taiwan in summer. Arrowheads in C indicate that the glabrous upper portion eventually decays or dies off in the senescing plant; **(J-K)** Magnification of the villous basal portion (arrows) in specimen from Small Port, KNP, southern Taiwan in winter; **(L)** Magnification of the villous basal portion (arrows) in specimen from Sail Rock, KNP, southern Taiwan in winter.

**Figure 3:**
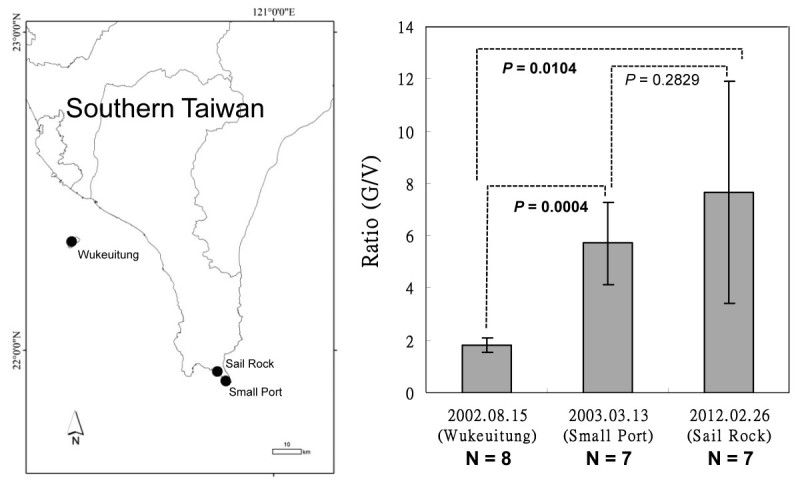
**Comparison of ratio between the height of the glabrous branch (G) and that of the villous branch (V) among samples from three different locations in southern Taiwan.** Map shows the three collection sites in southern Taiwan. Bars indicate standard deviation with eight different biological replicates at Wukeuitung and seven different biological replicates at Small Port and Sail Rock. The function “t.test” in the statistical software R was used to test if there is any significance of the G/V ratio among the three different locations.

**Figure 4:**
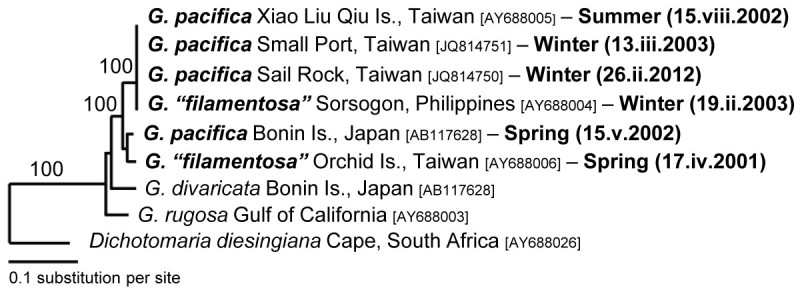
**Maximum-likelihood of phylogenetic tree of different specimens in the** ***G. pacifica*** **assemblage with** ***D. diesingiana*** **as the outgroup.** Seasonality for each specimen in the *G. pacifica* assemblage is highlighted in bold. The analyzed matrix merely includes 669 shared characters (i.e., nucleotides) due to the incomplete *rbc* L sequences for the specimen from Small Port, KNP, and southern Taiwan. Statistical supports are shown on branches. The value is 100 replicates of bootstrapping proportion values (> 50%) using ML analyses.

After we determining that *G. pacifica* and *G. “filamentosa”* are the same species, this study observed that specimens from different locations in southern Taiwan showed a range of external morphological variation in different seasons. Figure 2 shows that the specimens from Xiao-Liu-Qiu Island collected in the summer often possess a tuft of larger and distinctly villous branches in the lower part of the thallus (Figure 2A-2C, 2G-2I). The overall size of the lower villous part of the thallus and the density of upper glabrous branches vary among different individuals within the population. Some individuals had few clusters of loosely dichotomous glabrous branches in the upper part of the thallus (Figure 2A) and a tuft of wider and larger villous branches in the lower part of the thallus (Figure 2G). Some individuals had few clusters of densely dichotomous glabrous branches in the upper part of the thallus (Figure 2B) and a tuft of narrower and smaller villous branches in the lower part of the thallus (Figure 2H). Interestingly, one of the specimens consisted solely of very few glabrous branches in the upper part of the thallus (Figure 2C) and some residuals of glabrous branches can still be seen attaching on the lower villous branches (arrowheads in Figure 2C, 2I). A careful examination of the glabrous branches on this particular specimen showed that most of them were old and showed numerous lesions (image not shown). This observation suggests that the glabrous branches might eventually decay or die off in summer and the villous branches might be retained for some time after the decay/die-off of the glabrous branches, often leading to its misidentification as *G. filamentosa* (imagine the scenario that the last cluster of glabrous branches and those residuals decay in Figure 2C). In contrast, the specimens from Small Port (Figure 2D-2E, 2J-2K) and Sail Rock (Figure 2F, 2L) collected in the winter showed a tuft of small villous branches in the lower part of the thallus. The size of the villous branches in the lower part of the thallus and the density of the glabrous branches in the upper part vary across different individuals within the overall local population. In some cases, involving presumably more mature individuals, plants show several clusters of densely glabrous branches in the upper part of the thallus (Figure 2D) and a tuft of small villous branches in the lower part (Figure 2J). In other cases, representing presumably younger individuals, plants possess few clusters of densely dichotomous glabrous branches in the upper part of the thallus (Figure 2E-2F) and a tuft of tiny (occasionally unnoticeable) villous branches (Figure 2K-2L). To better quantify the external morphological difference among the specimens from different locations, we estimated the ratio between the height of the glabrous branch (G) and the villous branch (V). The G/V ratio of the specimens from Xiao-Liu-Qiu Island in summer is significantly smaller than those from Small Port and Sail Rock collected in the winter based on our comparison (Figure 3; *p* < 0.05). This result supports our previous observations that the specimens in the summer have larger villous branches than those in the winter. To rule out the possibility that our observed external variation results from the comparison of two different species, we additionally obtained *rbc* L sequences of the specimens from Small Port and Sail Rock and then compared these with that from Xiao-Liu-Qiu Island, as well as that from Sorsogon, Philippines. Results revealed that they share 100% identical *rbc* L sequences, indicative of conspecificity. Phylogenetic analysis also supports the same conclusion as they are grouped together (Figure 4). However, the inter-specific relationship differs from previous results. The lack of statistical support suggests that a difference might be caused by insufficient informative sites from the smaller set of characters (669 vs. 1407) used in this analysis (Figure 4). Because the glabrous branches of *G. pacifica* contain considerable mucilage, it is often difficult to obtain pure DNA for further PCR reactions. The traditional CTAB method (Doyle and Dickson, 1987) with at least three rounds of chloroform: isoamyl alcohol (24:1) treatments works more effectively than the commercial DNA extraction kit. Morphological observations and molecular analyses revealed that the villous branches are small in the winter and grow larger in the summer. Considering that one of the specimens from Xiao-Liu-Qiu Island showed extremely scarce glabrous branches (Figure 2F), it is tempting to speculate that the material identified as *G. filamentosa* might be the remaining villous part of senescing *G. pacifica*. Similar gross morphology and G/V ratio among specimens obtained during similar seasons over different years (e.g., Figure 2D-2E from March in 2003 and Figure 2F from February in 2012) (Figure 3) suggests that environmental cues such as temperature might serve as the most significant factor affecting external morphological development in *G. pacifica*.

### Morphological description

*Galaxaura pacifica* Tanaka, 1935: 55-57, Figures 5A, 5B, 6, pl. 17: Figure 2.

**Figure 5:**
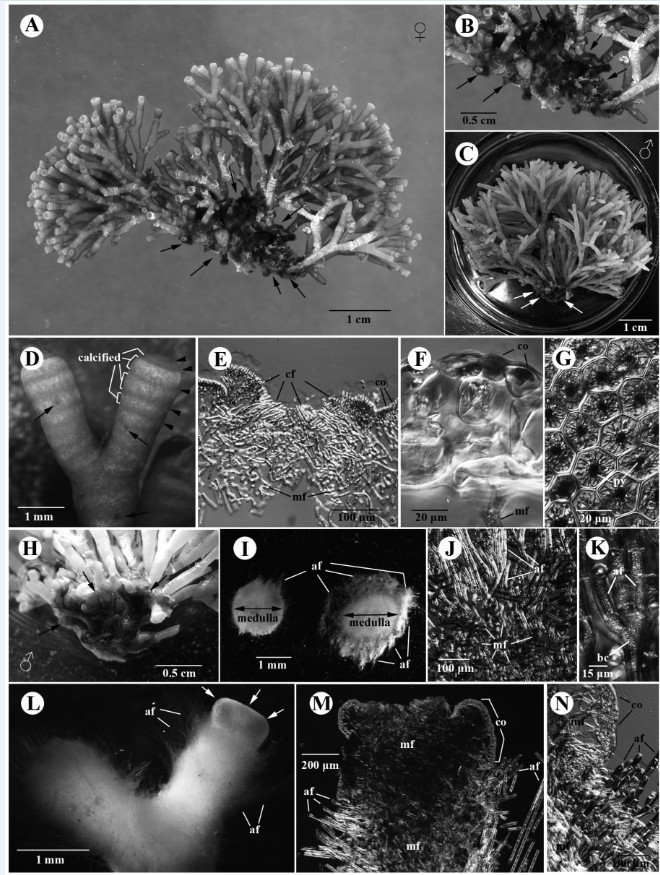
**External morphologies and cortical structures of glabrous-type thalli of** ***Galaxaura pacifica*** **Tanaka. (A-B, D-G)** Female gametophyte from Small Port. **(C, H-N)** Male gametophyte from Small Port. **(A)** Voucher specimen of female glabrous-type thallus for DNA analysis. Arrows indicate the basal villous branches in the lower portion of thallus; **(B)** Magnification of the villous basal portion (arrows); **(C)** Specimen of male glabrous-type thallus showing small villous basal portion (arrows); **(D)** Tip of the branch of the glabrous-type thallus showing annulations that are caused by the alternation of the calcified cortical regions and less calcified cortical regions (arrowheads). Reproductive structures are located throughout the glabrous branches (arrows); **(E)** Longitudinal section of the tip of the glabrous branch showing the sunken growing point with a cluster of slender cortical filaments (cf). Subsequently, these cortical filaments develop outward to form a layer of 3-celled cortex (co). The medullary filaments (mf) remain undifferentiated; **(F)** Cross section of the upper branch of female glabrous branch showing cellular cortex (co) and medullary filament (mf); **(G)** Surface view of cortical cells showing a stellate chromatophore with a pyrenoid (py); **(H)** Magnification of the villous basal portion (arrows); **(I)** Cross sections showing the comparison between lower part of the villous branch and the upper part of the villous branch. The upper part of the villous branch comprises of longer assimilatory filaments (af) whereas the lower part of the villous branch comprises of few assimilatory filaments (af); **(J)** Cross section of the villous basal portion showing that numerous assimilatory filaments (af) arise from a mass of medullary filaments (mf); **(K)** Assimilatory filaments (af) issued from undifferentiated and non-tumid basal cell (bc). **(L)** The glabrous branch (arrows) issued from the villous branch that comprises of numerous assimilatory filaments (af); **(M)** Longitudinal section of the transition zone between the glabrous branch and the villous branch showing that cortical structure transforms from assimilatory filaments (af) to cellular cortex (co). Medullary filaments (mf) are denser in the villous branch than that in the glabrous branch; **(N)** The magnification of the transition between the glabrous branch and the villous branch showing assimilatory filaments (af) from the villous branch and cellular cortex (co) from the glabrous branch. Noted that medullary filaments (mf) are very dense in the villous branch.

(Figures 5A-5N, 6A-6E, 7A-7C, 8A-8H, 9A-9E and 10A-10G).

**Figure 6:**
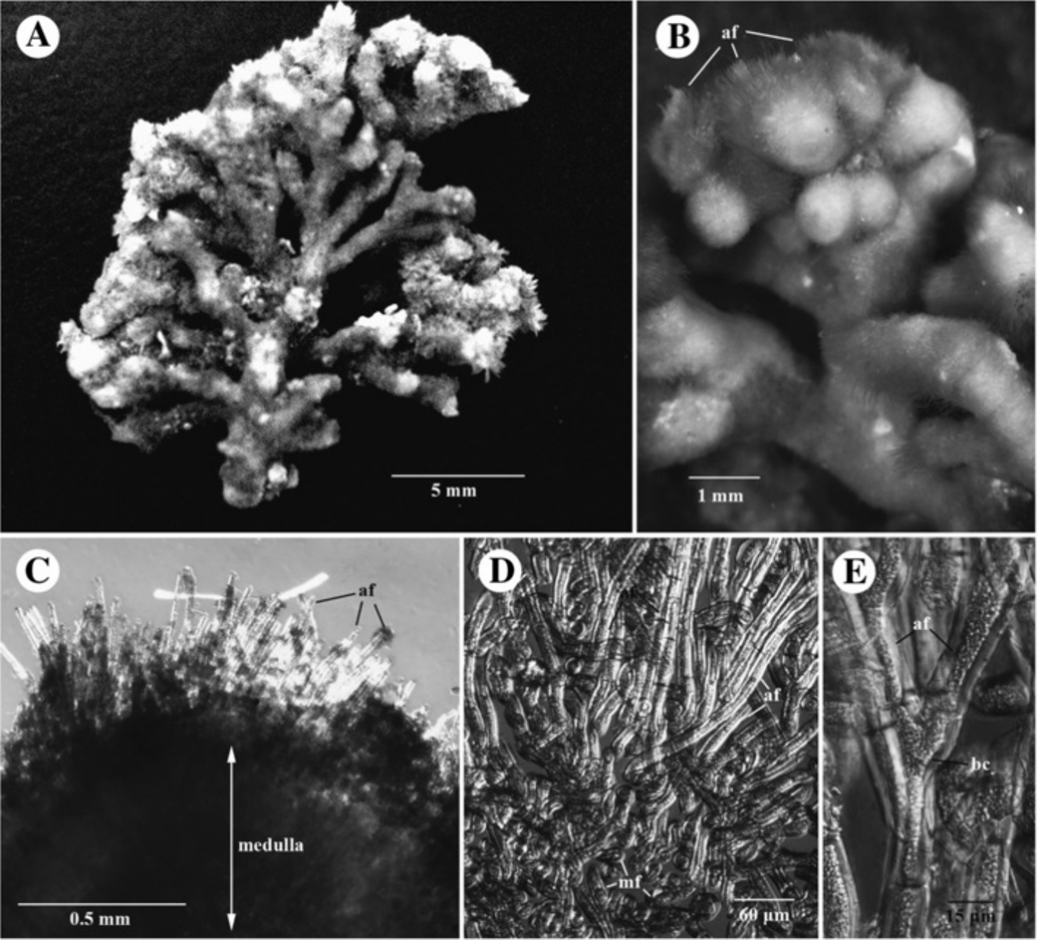
**The external morphologies and cortical structures of villous-type thalli in** ***Galaxaura pacifica*** **Tanaka.** (**A**) Voucher specimen of the villous plant from Bulusan, Sorsogon, the Philippines used for molecular analysis; (**B**) The magnification of the tip of branches showing a tuft of short villous branches with numerous assimilatory filaments (af); (**C**) Cross section showing two different layers, a cortical layer consisting of number long assimilatory filaments (af) and a medulla layer comprising of numerous dense medullary filaments; (**D**) Cross section showing long assimilatory filaments (af) arising from a mass of medullary filaments (mf); (**E**) Assimilatory filaments (af) issued from undifferentiated and non-tumid basal cell (bc).

**Figure 7:**
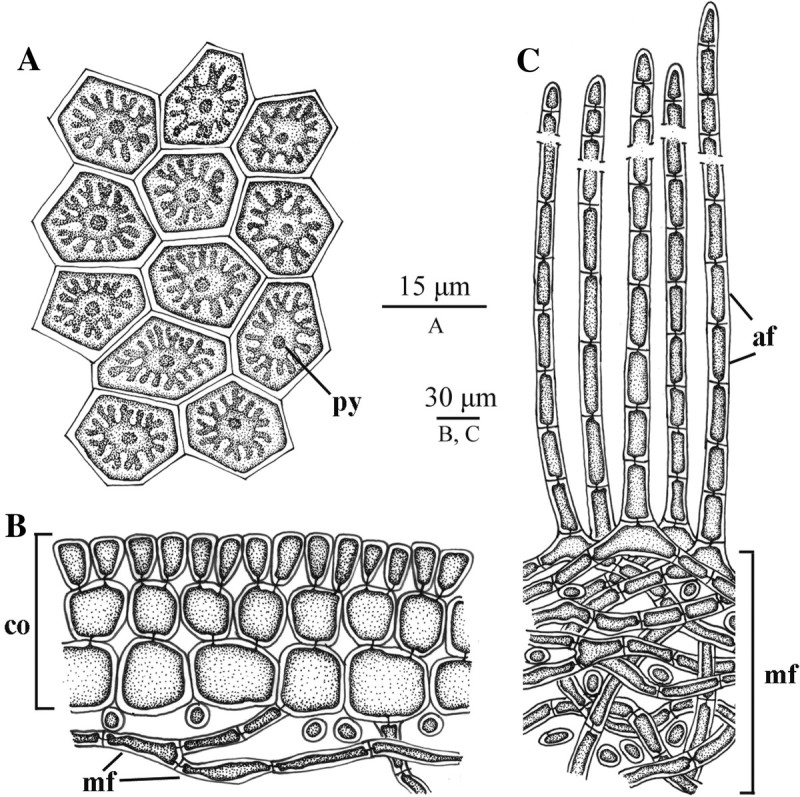
**Drawings of cortical structures of glabrous-type thalli of** ***Galaxaura pacifica*** **Tanaka.** **(A-C)** Female gametophyte from Small Port. **(A)** Surface view of cortical cells showing a stellate chromatophore with a pyrenoid (py); **(B)** Cross section of upper portion of glabrous branch showing cellular cortex (co) and medullary filament (mf); **(C)** Cross section of lower part of villous branch showing that assimilatory filaments (af) arise from a mass of medullary filaments (mf).

**Figure 8:**
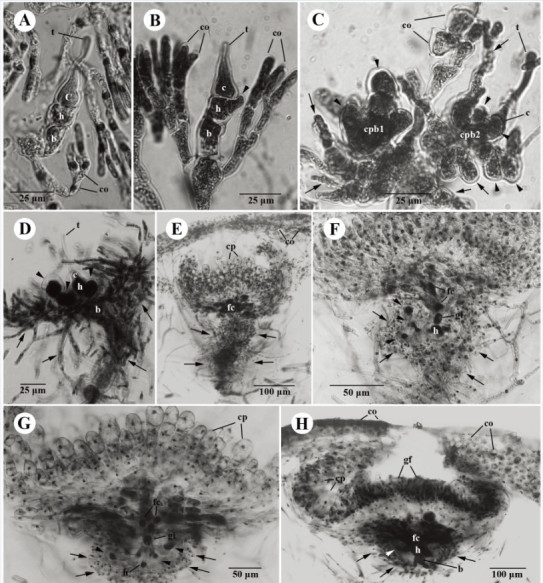
**Developmental sequence of the cystocarp of** ***Galaxaura pacifica*** **Tanaka from Small Port, KNP, southern Taiwan.** **(A)** Young carpogonial branch replaces one of the dichotomous vegetative cortical (co) branches showing a trichogyne (t), a carpogonium (c), a hypogynous cell (h), and a basal cell (b); **(B)** Young carpogonial branch protruding between the dichotomous vegetative cortical (co) branches showing a trichogyne (t), a carpogonium (c), a hypogynous cell (h) bearing the sterile branch (arrow), and a basal cell (b); **(C)** Two mature carpogonial branches borne on the vegetative cortical (co) branch showing trichogyne (t), carpogonium (c), hypogynous cell bearing several sterile branches (arrowheads), and basal cell bearing several involucral filaments (arrows); **(D)** Mature carpogonial branch showing a trichogyne (t), a carpogonium (c), a hypogynous cell (h) bearing several sterile branches (arrowheads) with darkly stained nuclei, and a basal cell (b) bearing several involucral filaments (arrows); **(E)** Cross section of young cystocarp showing developing gonimoblast filaments (gf), a distinct fusion cell (fc), a hypogynous cell (h), and a basal cell (b) bearing numerous involucral filaments (arrows) surrounding the base of cystocarp; **(F)** Cross section of immature cystocarp showing the fusion cell (fc) incorporated with the inner three gonimoblast cells and gonimoblast initial (gi), a hypogynous cell (h) bearing several modified sterile branches (arrowheads), and a basal cell (b) producing several involucral filaments (arrows) surrounding the base of the cystocarp; **(G)** Further development of immature cystocarp showing the gonimoblast filaments producing terminal carposporangia (cp), the distinct fusion cell (fc) incorporated with 7-10 inner gonimoblast cells and gonimoblast initial (gi), a hypogynous cell (h) bearing several modified sterile branches (arrowheads), and a basal cell bearing numerous involucral filaments surrounding the base of the cystocarp; **(H)** Cross section of mature cystocarp showing gonimoblast filaments (gf) producing terminal carposporangia (cp), the distinct fusion cell (fc), a hypogynous cell (h) with its derived sterile branch (arrowhead), and a basal cell (b) producing numerous involucral filaments (arrows) surrounding the base of the cystocarp.

**Figure 9:**
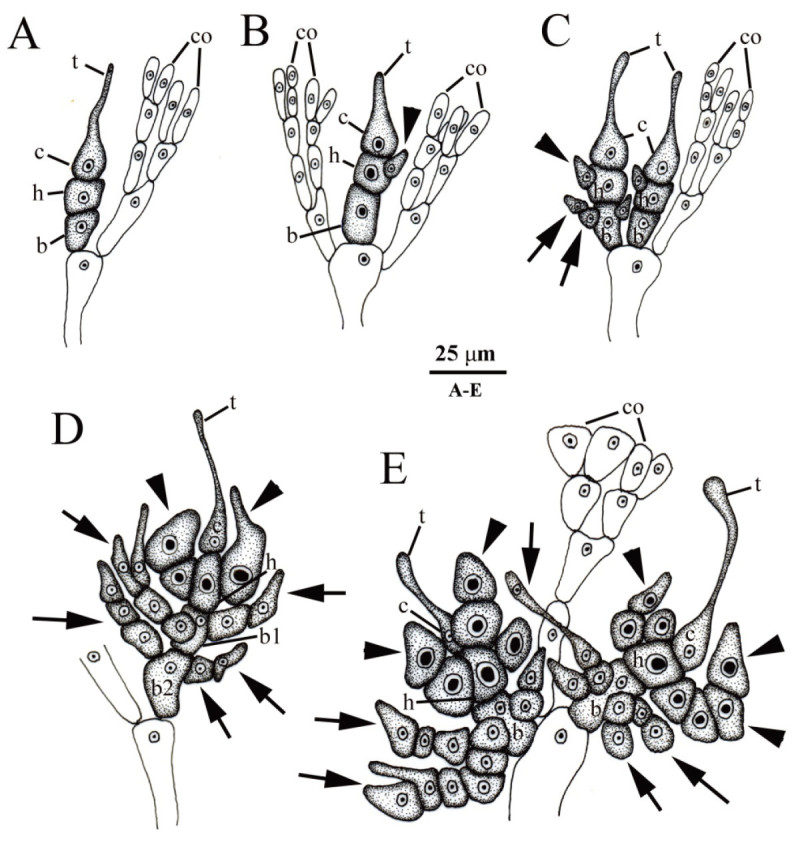
**Drawings of carpogonial branches of** ***Galaxaura pacifica*** **Tanaka from Small Port, KNP, southern Taiwan.** **(A)** Type I carpogonial branch pattern showing that the carpogonial branch initiation replaces one of cortical filaments (co). The carpogonial branch consists of the carpogonium (c) with the trichogyne (t), the hypogynous cell (h), and the basal cell (b); **(B)** Type II carpogonial branch pattern showing that the carpogonial branch initiation arises between cortical filaments (co). The carpogonial branch consists of the carpogonium (c) with the trichogyne (t), the hypogynous cell (h) bearing the sterile cell (arrowhead), and the basal cell (b); **(C)** Type III carpogonial branch showing a mixture of type I and type II carpogonial branch pattern. The carpogonial branch consists of the carpogonium (c) with the trichogyne (t), the hypogynous cell (h) bearing the sterile cell (arrowheads), and the basal cell (b) bearing several sterile filaments (arrows); **(D)** Developed type I carpogonial branch showing the carpogonium (c) with trichogyne (t), the hypogynous cell (h) bearing several sterile cells (arrowheads), and two basal cells (b1, b2) producing many sterile filaments (arrows); (**E**) Developed type III carpogonial branch showing the carpogonium (c) with trichogyne (t), the hypogynous cell (h) bearing several sterile cells (arrowheads), and the basal cell (b) producing many sterile filaments (arrows).

**Figure 10:**
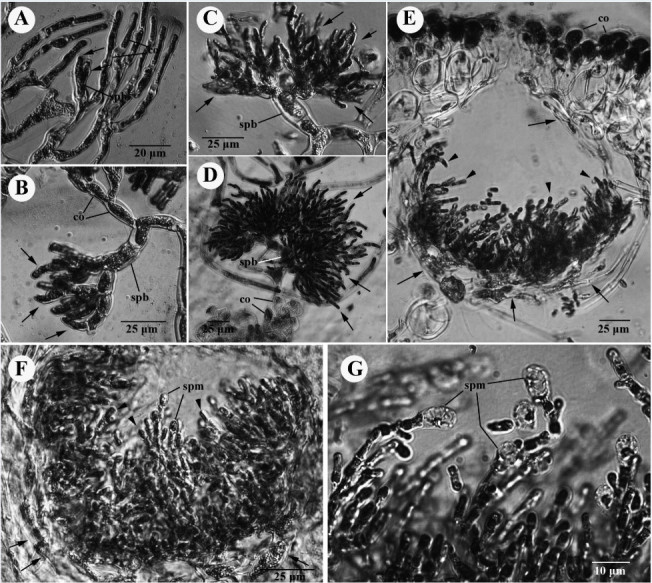
**Developmental sequence of the male structure of** ***Galaxaura pacifica*** **Tanaka from Small Port, KNP, southern Taiwan.** **(A)** Young spermatangial branch (spb) replaces one of the dichotomous vegetative cortical (co) branches bearing few primary spermatangial filaments (arrows); **(B)** Young primary spermatangial filaments further bearing several spermatangial filaments (arrows) at early stage of the spermatangial development; **(C)** Highly branched spermatangial filaments (arrows) produced from the spermatangial branch (spb) at a younger stage of spermatangial development; **(D)** Numerous spermatangial filaments (arrows) issued from spermatangial branch (spb) during spermatangial development; **(E)** Cross section of immature spermatangial conceptacle showing primary (arrows) and secondary (arrowheads) spermatangial filaments; **(F)** Cross section of mature spermatangial conceptacle showing primary (arrows) and secondary (arrowheads) spermatangial filaments, as well as the spermatangial mother cell (spm) terminally produced from the secondary spermatangial filaments; **(G)** Magnification of spermatangial mother cell (spm) terminally produced from the secondary spermatangial filaments.

#### Putative synonym

*Galaxaura filamentosa* Chou 1945: 39-41, pl. I: Figures 1, 2, 3, 4, 5 and 6; pl. VI: Figure 1 (type locality: Sulphur Bay, Clarion Island, Revillagigedo Islands, Mexico).

#### Type locality

Bonin Islands, Japan.

**Distribution**: predominately distributed in the warm temperate, subtropical, and tropical regions of the Pacific Ocean, including Japan, Taiwan, the Philippines, and possibly the Revillagigedo Islands of Mexico.

#### Specimens examined

Xiao-Liu-Qiu Island, southern Taiwan: (1) Wukeuitung, coll. S.L. Liu and C.S. Lin, 15.viii.2002 (#TU_GaPa2002.08.15.01 ~ #TU_GaPa2002.08.15.08, female gametophyte); Kenting National Park, southern Taiwan: (1) Small Port, coll. S.L. Liu, 13.iii.2003 (#TU_GaPa2003.03.13.01 ~ #TU_GaPa2003.03.13.03, female gametophyte); (2) Small Port, coll. S.L. Liu, 13.iii.2003 (#TU_GaPa2003.03.13.04 ~ #TU_GaPa2003.0312.07, male gametophyte); (3) Sail Rock, coll. S.L. Liu, 19.ii.2012 (#TU_GaPa2012.02.19.01 ~ #TU_GaPa2012.02.19.07, female gametophyte); Sorsogon, the Philippines: (1) Bulusan, coll. L.M. Liao and S.L. Liu, 19.ii.2003 (#TU_GaPa2003.02.19.01, possible remnants of the villous parts of senescing gametophyte or tetrasporophyte).

#### Habitat and seasonality

Collections were made seasonally in February, March, and August. Plants often grew on rocky (or coral reef) substrates at depths of 1-3 m.

#### Habit and vegetative structure

The thalli comprise two distinct forms: 1) glabrous-type individual (Figure 5A, 5C) and 2) villous-type individuals (Figure 6A). Glabrous-type thalli are light-red or pink in color and up to 6 cm high at full maturity. There are no gross morphological differences between female (Figure 5A) and male plants (Figure 5C). Villous-type thalli are dark-red in color and up to 2.5 cm in height. Both types of thalli initially consist of a primary cylindrical axis that originated from a discoid holdfast. The holdfast diameter is approximately 1-3 mm. The initial primary terete axis continuously develops several terete branches. These branches are produced in a dichotomous or subdichotomous manner. The length of internodes is 5-15 mm, and the width of branches is 1-2 mm. Branches in the glabrous-type thallus are smooth in the upper portion (Figure 5A, 5C) and villous in the lower portion of the thalli (Figure 5B, 5H), whereas the branches in the villous-type thallus are hairy throughout (Figure 6A). Both types of thalli show light to heavy calcification in the cortical and medullary parts. Different cortical sections on the terminal branches of the glabrous area show different degrees of calcification that subsequently lead to the appearance of annulations (Figure 5D). The cross section of the branches from the glabrous-type plants (i.e., gametophytes) and that from the villous-type plants display two different cortical structures. The first type can be observed from the smooth portion of the glabrous-type thallus (Figure 5E-5F) wherein growth is apical with a sunken growing point (Figure 5E). Young cortical initials on the apex of glabrous branches are slender (Figure 5E) and then develop into a three cell-layers (Figures 5F, 7B). The outermost layer consists of highly pigmented epidermal cells 12-18 (20) μm in diameter (Figures 5F, 7B). The middle layer is composed of slightly larger cells that are 25-38 μm in width and 30-40 μm in length (Figures 5F, 7B). The innermost layer consists of the largest cells which are 25-50 μm in width and 38-105 μm in length (Figures 5F, 7B). In the surface view the outermost cortical cells show 4-6 sided and angular cells. Each cell contains one well-developed stellate chromatophore with a large central pyrenoid (Figures 5G, 7A). The second cortical type can be observed from the villous branches of the lower section of the glabrous-type thalli (Figure 5B, 5H, 5L) and the villous-type thalli (Figures 6A-6B). The cross-sectioned inner part of the branch is the medulla (Figures 5I, 6C, 7C), which comprises heavily calcified dense medullary filaments (Figure 5I). The inner layer of the cortex shows a mixture of medullary and assimilatory filaments (Figures 5J, 6D, 7C). The outer layer of the cortex is composed of 10- to 50-celled, long assimilatory filaments (Figures 5I-5J, 6C-6D) that arise from an undifferentiated, non-swollen basal cell (Figures 5K, 6E). These long assimilatory filaments are dense and up to 1 mm long at distal portions of villous branches, but are scarce at the lower part of villous branches (Figures 5H, 6B). Overall, there are no obvious differences in the cortical structures between the villous branches in the lower part of the glabrous-type thalli and the villous-type thalli. The assimilatory filament growth pattern of *G. pacifica* differs from that in the villous-type thalli of *G. rugosa*, which possessed two different kinds of assimilatory filaments: long (5- to 12-celled) and short (2- to 3-celled) assimilatory filaments (for details, see Figure 6d in Wang et al. 2005). The swollen basal cell from which the assimilatory filaments of *G. rugosa* are derived was not observed in the villous branches of *G. pacifica*. For the glabrous-type thalli, young glabrous branches are issued from the tip of villous branches (Figure 5L). The cortical structure transforms from the early production of assimilatory filaments to the subsequent production of a 3-celled cortex (Figure 5M-5N). Following transformation, the medullary filaments become less compact in the glabrous branch compared to those in the villous branch (Figure 5M-5N).

#### Reproductive structure

Tetrasporangia were not observed in our materials. Plants are dioecious. All reproductive structures are scattered throughout the glabrous branches (Figure 5D) and located in the boundary between the cortex and the medulla. Both female (i.e., cystocarp) and male structures develop to form a conceptacle at maturity.

Cystocarps are scattered over the fertile thallus except for the basal part of the glabrous branches (Figure 5D). During cystocarp development, young carpogonial branches are often 3-celled and consist of the carpogonium, the hypogynous cell, and the basal cell (Figures 8A, 9A). A 4-celled carpogonial branch with one additional basal cell is occasionally observed in our materials (Figure 9D). Three different types of carpogonial branch initiation were observed near the branch tip. The first type replaces an ordinary vegetative filament (Figures 8A, 9A). The second type arises between the dichotomous ordinary filaments (Figures 8B, 9B). The third type is a combination of the first and the second types of growth patterns (Figures 8C, 9C). When carpogonial branches of the third type becomes fertilized, neighboring carpogonial branches stop developing. However, fate of these abortive carpogonial branches remains unclear. Before fertilization, three to four sterile branches arise from the hypogynous cell (Figures 8C, 9D-9E). As the carpogonial branch matures, the hypogynous cell and its derived sterile branches enlarge and become darkly stained (Figures 8D, 9D-9E). Their single nuclei become extremely dark. The basal cell cuts off several involucral filaments from the cells, the nuclei of the basal cell and its derived filaments do not enlarge (Figures 8D-8F, 9D-9E). Fertilization was not observed, but the gonimoblast initial is presumably produced from the fertilized carpogonium. Pit plugs linking the inner gonimoblast cells to the gonimoblast initial break down at an early stage of carposporophyte development (Figure 8E-8F), but the hypogynous cell, the cells of the sterile branches, and the basal cell still remain distinct and retain their relative positions throughout cystocarp development (Figure 8E-8F). The involucral filaments from the basal cell do not form the pericarp and are restricted to the base throughout the development of the cystocarp (Figure 8D-8F). Ultimately, seven to ten inner cells of the gonimoblast filaments and the basal gonimoblast cell are incorporated into a multinucleate fusion cell in the mature carposporophyte (Figure 8E-8G). Mature cystocarps reach 400-800 μm in diameter (Figure 8H). The secondary gonimoblast filaments derived from the primary gonimoblast filaments produce terminal oval to obovate carposporangia, 12-45 μm by 38-80 μm (Figure 8G-8H). New carpospores sometimes arise from the remnant walls of previously shed carposporangia.

During development of male structure, the spermatangial branch initial arises in place of one of the cortical filaments (Figure 10A). Subsequently, the young spermantangial branch divides laterally or transversely to give rise to primary spermantangial filaments (Figure 10B). These primary spermatangial filaments further divide to produce numerous spermantangial filaments (Figure 10C) that grow into a cluster of highly branched spermantangial filaments (Figure 10D). Eventually, they form a hemispherical conceptacle (Figure 10E), 180-240μm in diameter at maturity (Figure 10F). Numerous secondary spermatangial filaments are issued from the inner side of the conceptacle and produce spermatangial mother cells terminally (Figure 10F). One spermatangium, 4-6 μm in width and 8-10 μm in length, is cut off terminally from the secondary spermatangial filament (Figure 10G).

#### Remarks

Tanaka (1936) and Tseng (1941) reported the occurrence of *Galaxaura rudis* Kjellman in Taiwan (as Formosa) and Hainan Island, respectively. Based on their descriptions, the specimens from these two localities showed undifferentiated non-tumid basal cells (i.e., supporting cells of assimilatory filaments). Thus, both authors argued that these specimens were morphologically distinct from *G. rudis*. Afterward, when Chou (1945) proposed the new species, *G. filamentosa*, she suggested that the specimens from Taiwan and Hainan Island should be treated as synonyms of *G. filamentosa* because their cortical structures are extremely similar. This study shows that the cortical structure of the specimen from the Philippines is highly similar to that of *G. filamentosa* from Taiwan and Hainan Island. The size of *G. filamentosa* from these three locations is small and ranges between 2 and 4 cm (more often less than 2.5 cm) in height. It is also noteworthy that Svedelius (1953) showed that the gross morphology of *G. filamentosa* from Hawaii comprises two different types, the tufted type and the freely growing type. Most tufted-type specimens were 1.5 cm high and crowded together. In contrast, the freely growing type could grow up to 3 cm in height. Consistent with the observations on *G. filamentosa* (as *G. rudis*) by Tanaka (1936) and Tseng (1941), most *G. filamentosa* specimens from Hawaii examined by Svedelius (1953) are “obtuse at the apex”. Svedelius (1953) also reported that *G. filamentosa* lacks the sunken growing point at the tip of branch and lacks fertile structures. This study shows that these unique morphological features may not be surprising when considering that *G. filamentosa* might be the remaining part of the tufted villous branches of *G. pacifica*. It will be interesting to test this hypothesis if *G. filamentosa*-type plants are more common in the tropical region of the Pacific Ocean while *G. pacifica*-type plants are more common in the subtropical or temperate region of the Pacific Ocean.

Although the specimen from the Philippines had a cortical structure similar to that of *G. filamentosa*, the type locality of *G. filamentosa* in Mexico (southeastern Pacific coast), is far from Taiwan, Hainan, and the Philippines (northwestern Pacific coast). Furthermore, the thallus size of *G. filamentosa* recorded in Chou (1945) [3.5-5 cm] is generally larger than our specimen from the Philippines, which was only 2.5 cm in height. Such a variation of plant size might be caused by ambient environmental factors such as light or temperature. However, without verification based on molecular analyses of *G. filamentosa* specimens obtained from its type locality, we can at best only propose that *G. filamentosa* might be the same species as *G. pacifica* from the northwestern Pacific Ocean.

## Discussion

The relationship of the four Galaxauraceae genera *Actinotrichia*, *Dichotomaria*, *Galaxaura*, and *Tricleocarpa* in our phylogenetic analyses is consistent with those of Huisman et al. (2004a), Wang et al. (2005), and Liu and Wang (2009). In the genus *Galaxaura*, it is noteworthy that many ambiguous taxa within the *G. rugosa* assemblage require further morphological examinations, particularly considering that only one species can be identified with certainty as *G. cuculligera* based on morphological characters (Kurihara et al., 2005).

This study provides the first evidence that two morphologically distinct species, *G. pacifica* and *G. filamentosa*, should be treated as a single species, *G. pacifica*. *Galaxaura pacifica* is characterized by having multi-axial thalli, glabrous, slightly rugose terminal branches, and densely villous branches in the lower part of the plant (Tanaka, 1935); the latter is a unique morphological trait (see the illustration of Figure 11 of Tanaka, 1936) that can be used to separate *G. pacifica* from the other species of the genus *Galaxaura*. In contrast, *G. filamentosa* is characterized by villous branches occurring throughout, stretching from the lower portion to the terminal portion of the thallus with only long assimilatory filaments shown in the cross section of its branches (Chou, 1945). Based on these characteristics, Chou (1945) considered it a new species. The evidence from our molecular analyses however revealed that these two species should be treated as the same species since their *rbc* L sequences were unexpectedly identical, consistent with the assumption made by Huisman and Borowitzka (1990). We propose to combine these two species as a single species, *G. pacifica,* with this name having taxonomic priority over *G. filamentosa.* From our morphological observations, *G. pacifica* is now characterized as (1) a glabrous or villous multi-axial plant with either glabrous or villous branches and a discoid holdfast; (2) glabrous branches with a three-celled layer of cortex; (3) villous branches with numerous long assimilatory filaments arising from the swollen basal cells; (4) three-celled carpogonial branches (consisting of a carpogonium, hypogynous cell, and basal cell) that are produced from the replacement of one of the dichotomous vegetative cortical filaments (referred to as type I), between the dichotomous vegetative cortical filament (referred to as type II), or the mixture of both type I and type II growth patterns (referred to as type III); (5) gonimoblast initial produced from the fertilized carpogonium; (6) involucral filaments derived from the basal cell of the carpogonial branch remaining at the base throughout cystocarp development; and (7) a distinct fusion cell derived from the fusion of 7-10 inner gonimoblast cells without the involvement of the hypogynous cell, the sterile branches from the hypogynous cell and the basal cell.

Two possible scenarios could explain the different morphological features in *G. pacifica*. First, the external morphology of *Galaxaura* species can be dimorphic (i.e., gametophyte and tetrasporophyte) during their life history (e.g., Huisman and Borowitzka, 1990;Huisman et al., 2004b;Kurihara et al., 2005;Wang et al., 2005). This dimorphic life history in *Galaxaura* was first observed by Howe (1917;1918), who noted that different life history stages (i.e., gametophyte vs. tetrasporophyte) in *Galaxaura* (at the time including *Dichotomaria*) can have two different external morphologies with different cellular structures (in *Galaxaura*) or same external morphology with two different cortical structures (in *Dichotomaria*) - a phenomenon referred to as dimorphism. Recently, dimorphism was supported by evidence from molecular analyses (Huisman et al., 2004b;Kurihara et al., 2005;Wang et al., 2005). Second, the villous plant might merely be an immature form of the gametophyte (Huisman, pers. comm.), although some studies have considered them as possible tetrasporophytic forms of *G. rugosa* (e.g., Chou, 1945;Papenfuss et al., 1982). Huisman and Borowitzka (1990) indicated some precautions with directly treating a villous thallus as a tetrasporophytic form of *Galaxaura*, particularly for specimens without any evidence of tetrasporangia. Indeed, no tetrasporangial structures have been found in *G.* “*filamentosa*” (= *G. pacifica*) from Taiwan (AY688006; Figure 1) and the Philippines (AY688004; Figure 1), consistent with the observations of Chou (1945) and Huisman and Borowitzka (1990). Svedelius (1953) and Huisman and Borowitzka (1990) indicated that the reproductive structures of *G. filamentosa* have never been found and its cortical structure is vegetatively identical to the hirsute (i.e., villous) basal portion of *G. rugosa*, suggesting that further work may show that *G. pacifica* might prove to be a synonym of *G. rugosa*. Thus, for the first time, this study provides evidence to further support these earlier proposals that *G. filamento* sa should be considered as the same species as *G. pacifica*. It should be noted that the species *G. rugosa* described in Huisman and Borowitzka (1990) may comprise several different species (including the *G. rugosa* assemblage and the *G. pacifica* assemblage) as shown by Wang et al. (2005) and this study. For example, the specimen in Figure 10 of Huisman and Borowitzka (1990) is externally similar with our materials in the *G. rugosa* assemblage, and that in Figure 11 of the same paper is externally similar to our materials comprising the *G. pacifica* assemblage. Thus, it is likely that the specimens with obvious villous basal portions cited by Huisman and Borowitzka (1990) (e.g., the specimen in Figure 11 of their paper) should be considered phylogenetically close to the *G. pacifica* assemblage. To verify the possibility whether the villous plant is a tetrasporophyte or not, a further study to investigate their ploidy levels should be required to clarify this issue, among others. According to the materials collected from Xiao-Liu-Qiu Island, Taiwan, the villous plants might be merely the remnants of the basal villous part of the thallus after the distal glabrous portions of the thallus die off in the summer. As also evidenced by *G. “filamentosa”* from Sorsogon, Philippines, this plant was collected from the warmer regions of the Pacific Ocean where water temperature is comparable to that in higher latitudes of the Pacific Ocean in the summer. Considering that we never saw the seasonal co-occurrence of *G. “filamentosa”* and *G. pacifica*, our observations therefore support the hypothesis that certain environmental cues (e.g., temperature) might stimulate the development of *G. filamentosa*-type plants from *G. pacifica*-type plants.

Previously, Papenfuss et al. (1982) proposedd that *G. pacifica* should be considered synonymous with *G. rugosa* because their morphological characters were not distinguishable. In contrast, Kurihara et al. (2005) found that *G. pacifica* could be separated from *G. rugosa* based on molecular evidence. Our results are consistent with their observations and further support that *G. pacifica* should be recognized as an independent species different from *G. rugosa*. Contrary to the observations by Papenfuss et al. (1982), we can morphologically separate *G. pacifica* from the other species of *Galaxaura* based on three morphological grounds: 1) obvious villous branches in the lower part of gametophytic plants, 2) long assimilatory filaments without swollen basal cells in the villous branches, and 3) three different growth patterns of carpogonial branches (explained in more details below).

Compared to other species of *Galaxaura*, we found that carpogonial branches of *G. pacifica* have an unusual growth pattern. Some carpogonial branches arose to replace one of the dichotomous terminal cortical filaments (type I) while some of them arose between the dichotomous terminal cortical filaments (type II). Occasionally, two carpogonial branches, one of which originated from type I and the other originated from type II, grew on the same terminal cortical filament, suggesting a mixture of type 1 and type 2 growth pattern of carpogonial branches (referred to as type III). After fertilization, only one of them could successfully develop into a carposporophyte. Once one of them was fertilized, the others seemed to stop their development and senesce subsequently. To quantify the frequency of these three different types of carpogonial branch growth patterns, we randomly observed 30 different carpogonial branches and found that the type I (25 out of 30) is the most common type of carpogonial branch growth pattern and the type II (3 out of 30) and the type III (2 out of 30) are less frequent. Previously, only type I carpogonial branch was observed in the genera *Dichotomaria*, *Galaxaura*, and *Tricleocarpa* (Huisman and Borowitzka, 1990;Wang et al., 2005b), whereas the type II carpogonial branch was only observed in the genus *Actinotrichia* (Wang and Chiang, 2001;Liu and Wang, 2009). Therefore, the ontogeny of carpogonial branches has been suggested as a useful character for separating different genera among the members of the family Galaxauraceae (e.g., Wang and Chiang, 2001;Liu and Wang, 2009). Considering that both types of carpogonial branches can be found in *G. pacifica*, the value of this character in separating genera is questionable. Instead, this mixture of growth pattern types of carpogonial branches might be a useful taxonomic feature at the species level to separate *G. pacifica* from other species in the genus *Galaxaura*.

Two caveats in this study should be addressed. The first is that the *rbc* L sequences of our specimens from Xiao-Liu-Qiu Island, Taiwan and Sorsogon, the Philippines genetically differ from the specimen obtained from the type locality of *G. pacifica*. In total, 27 nucleotides of *rbc* L sequence (approx. 2%; uncorrected *P-* distance) were found to be different between these two populations. Such a sequence divergence exceeds the species-level boundary (< 0.5%), which was delineated based on previous *rbc* L sequence analyses (e.g., Kurihara et al., 2005). Compared to morphological descriptions of gametophytic plants of *G. pacifica* in Tanaka (1935), we could not however recognize any morphological difference between these two entities. Furthermore, we failed to recognize any morphological differences between the two genetically different specimens under study, which were initially identified as *G.* “*filamentosa*” (AY688006 and AY688004 in Figure 1). Both specimens show numerous long assimilatory filaments without a swollen basal cell, and their external morphological features cannot be distinguished (unpublished data). *Rbc* L sequences between these two specimens differed by about 2.6%. Overall, our results implied that morphological traits within the *G. pacifica* assemblage vary in a subtle manner despite their genetic heterogeneity. The species definition could be challenging in the light of taxonomic studies, particularly when the available evidence is limited. Without examining if a cross between any two populations of *G. pacifica* can produce viable hybrids or if there is any gene flow among them, the species boundary in *G. pacifica* will remain unresolved and subject to continuous disputes. Therefore, based on our limited information, the typological species concept, which is considered a conservative approach (e.g., Kurihara and Huisman, 2006), was applied to our case since no morphological difference can be found among the *G. pacifica* assemblage to convincingly argue for its distinctness. The second caveat is that *G. filamentosa* may be still a valid species since as yet no one has obtained *rbc* L sequences from materials collected from the type locality in the Revillagigedo Islands, Mexico. Further work may provide additional evidence supporting its synonymy with *G. pacifica*.

Clearly, the morphological and reproductive structures in *Galaxaura* show many similarities and can be sources of confusion. Collective evidence from a combination of approaches including molecular analyses, population genetic analyses, and detailed morphometric comparisons will be required to unequivocally delineate the species-level boundary of *Galaxaura* in the future. This attempt to use molecular tools for the elucidation of relationship between two morphologically different (e.g., gametophytic vs. tetrasporophytic) species of the calcified red algal family Galaxauraceae is only beginning to scratch the surface of this taxonomic puzzle. So far, only two cases have been argued. The first example is the pair of *G. rugosa* and *G. subverticillata* (= *G. rugosa*) on the basis of large subunit rDNA sequence analysis (Huisman et al., 2004b). The second example is the pair of *D. apiculata* and *D. hystrix* (= *D. apiculata* ) based on *rbc* L and ITS sequence analyses (Kurihara et al., 2005). Thus, our study provided another example in attempting to match two morphologically different species (from the northeast Pacific Ocean), *G. pacifica* and *G. filamentosa*, and to recognize them as a single species, *G. pacifica*. Since numerous species have been historically established in the genus *Galaxaura* and *Dichotomaria* based largely on their cortical structures (e.g., Kjellman, 1900), the biodiversity of the genus *Galaxaura*, as well as the genus *Dichotomaria*, was definitely overestimated in previous studies (i.e., Kjellman, 1900). Our study provides another example of documented dimorphism in different stages of the life history within a single species of *Galaxaura* (first proposed by Howe, 1917,1918), and sheds light on the biodiversity and unique biology of the calcified red algal genus *Galaxaura*.

## Authors’ original submitted files for images

Below are the links to the authors’ original submitted files for images. Authors’ original file for figure 1 Authors’ original file for figure 2 Authors’ original file for figure 3 Authors’ original file for figure 4 Authors’ original file for figure 5 Authors’ original file for figure 6 Authors’ original file for figure 7 Authors’ original file for figure 8 Authors’ original file for figure 9 Authors’ original file for figure 10
