# Supplementary figures and images for: Conspecificity of two morphologically distinct calcified red algae from the northwest Pacific Ocean: Galaxaura pacifica and G. filamentosa (Galaxauraceae, Rhodophyta)
Source: Bot Stud. 2013 Jul 18;54:1. doi: 10.1186/1999-3110-54-1 (PMC5383922; doi:10.1186/1999-3110-54-1)

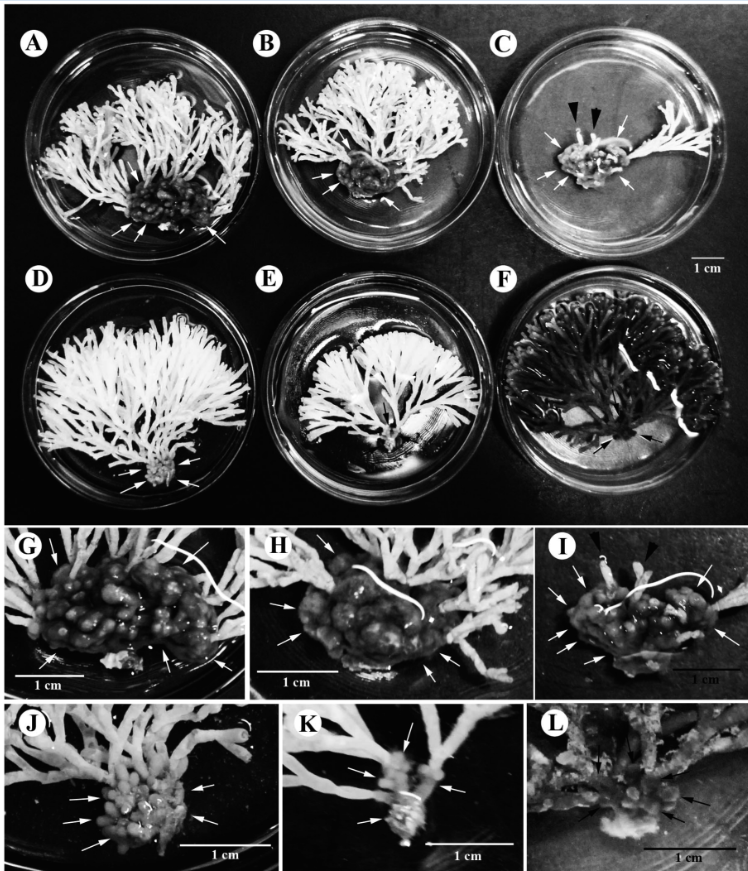

Supplement: Supplementary file 2 — Authors’ original file for figure 2 [file 40529_2011_2_MOESM2_ESM.png]

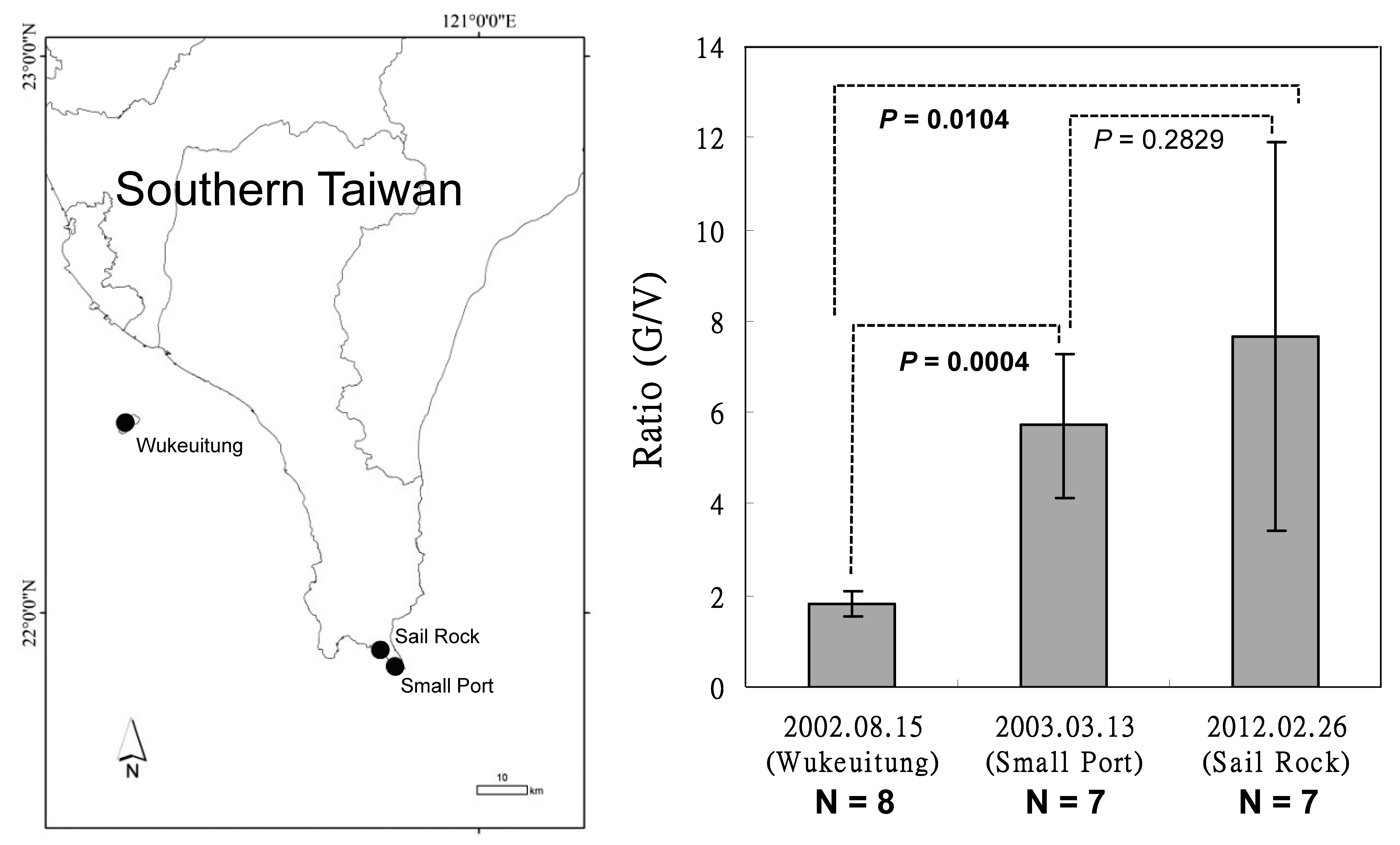

Supplement: Supplementary file 3 — Authors’ original file for figure 3 [file 40529_2011_2_MOESM3_ESM.tiff]

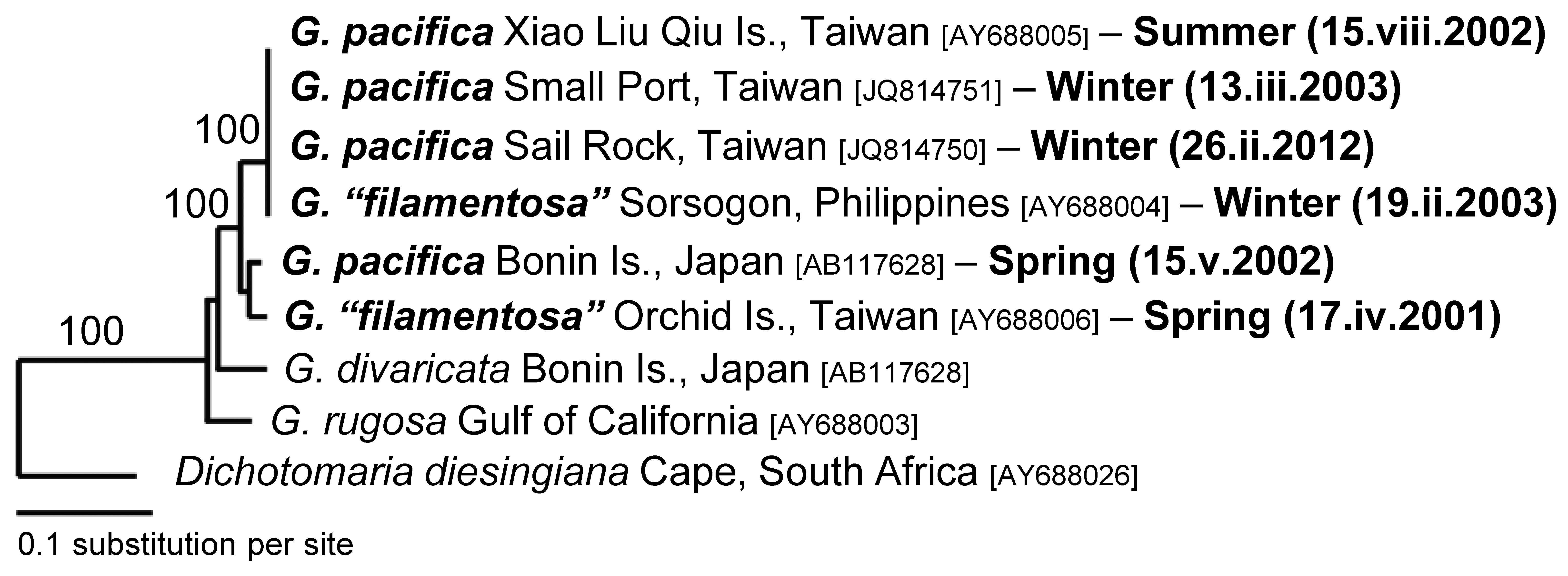

Supplement: Supplementary file 4 — Authors’ original file for figure 4 [file 40529_2011_2_MOESM4_ESM.tiff]

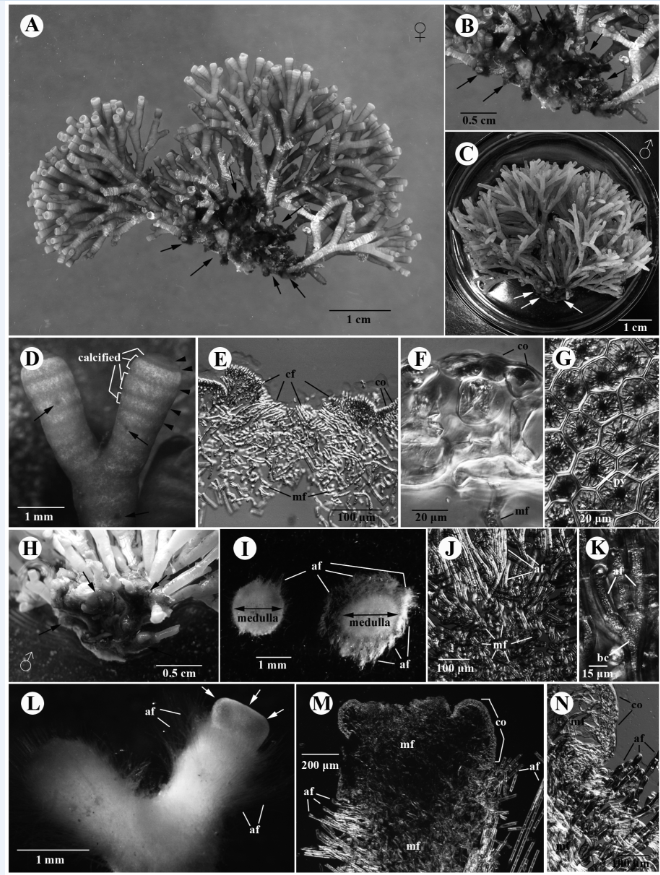

Supplement: Supplementary file 5 — Authors’ original file for figure 5 [file 40529_2011_2_MOESM5_ESM.png]

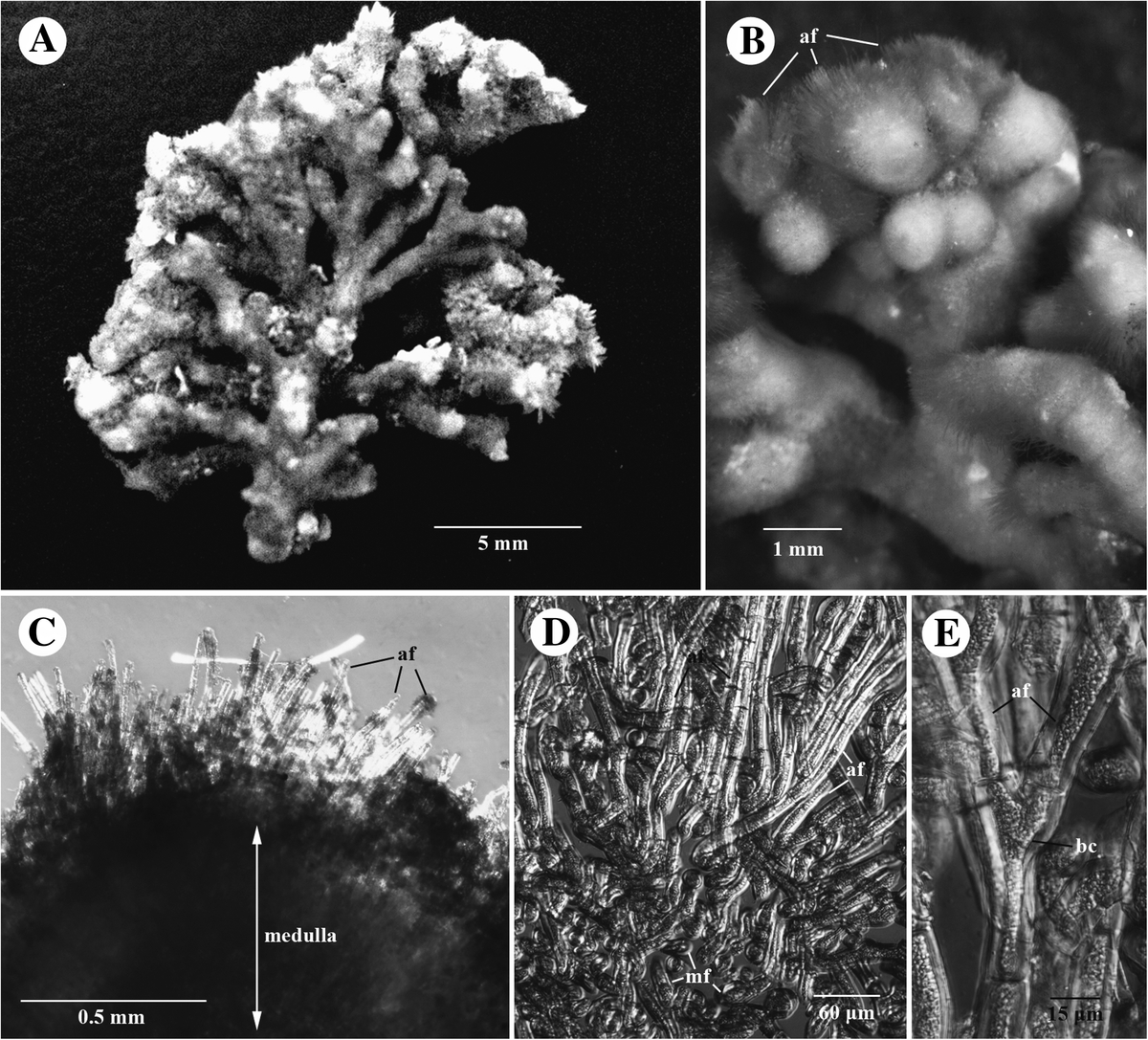

Supplement: Supplementary file 6 — Authors’ original file for figure 6 [file 40529_2011_2_MOESM6_ESM.jpeg]

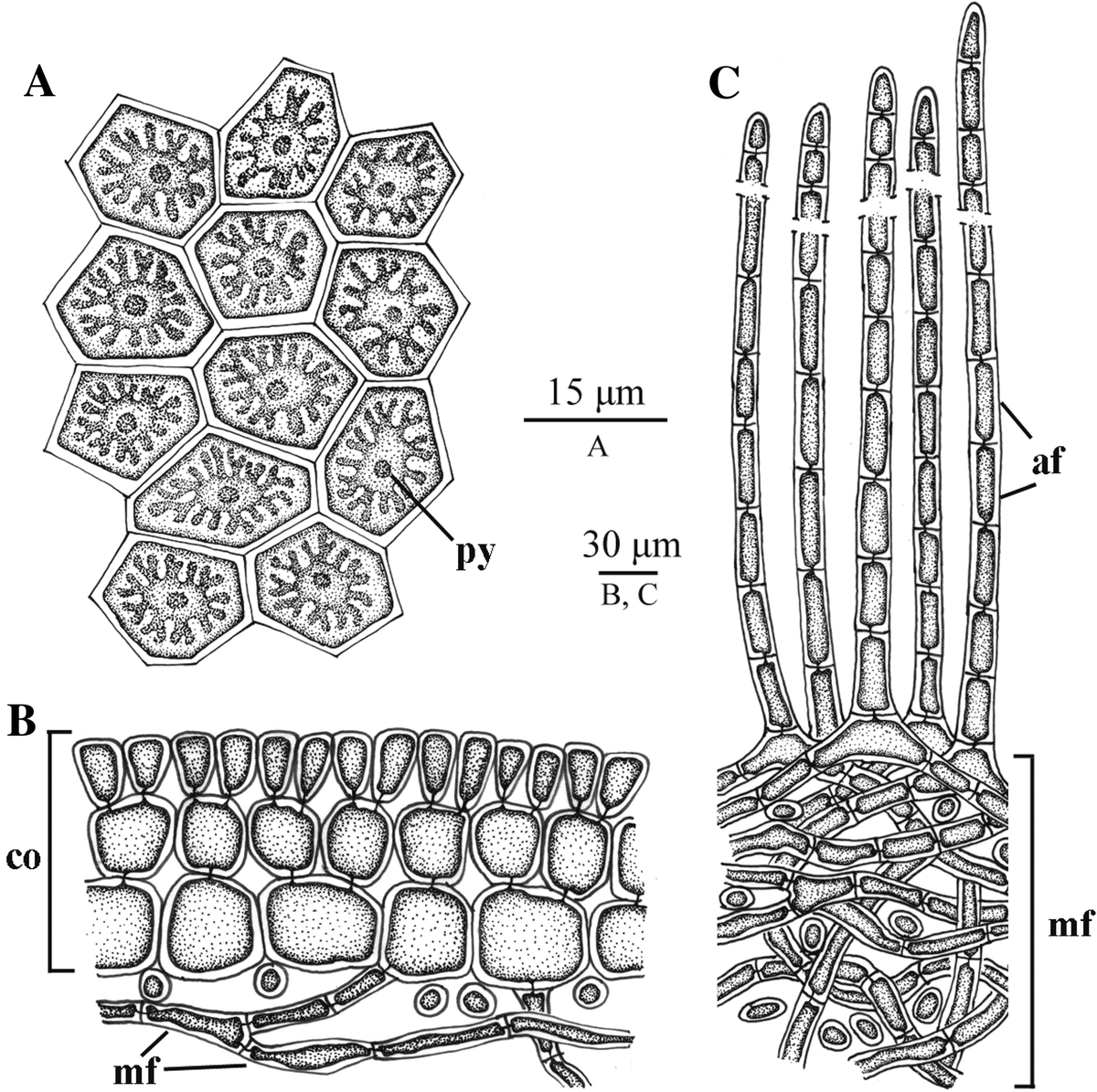

Supplement: Supplementary file 7 — Authors’ original file for figure 7 [file 40529_2011_2_MOESM7_ESM.jpeg]

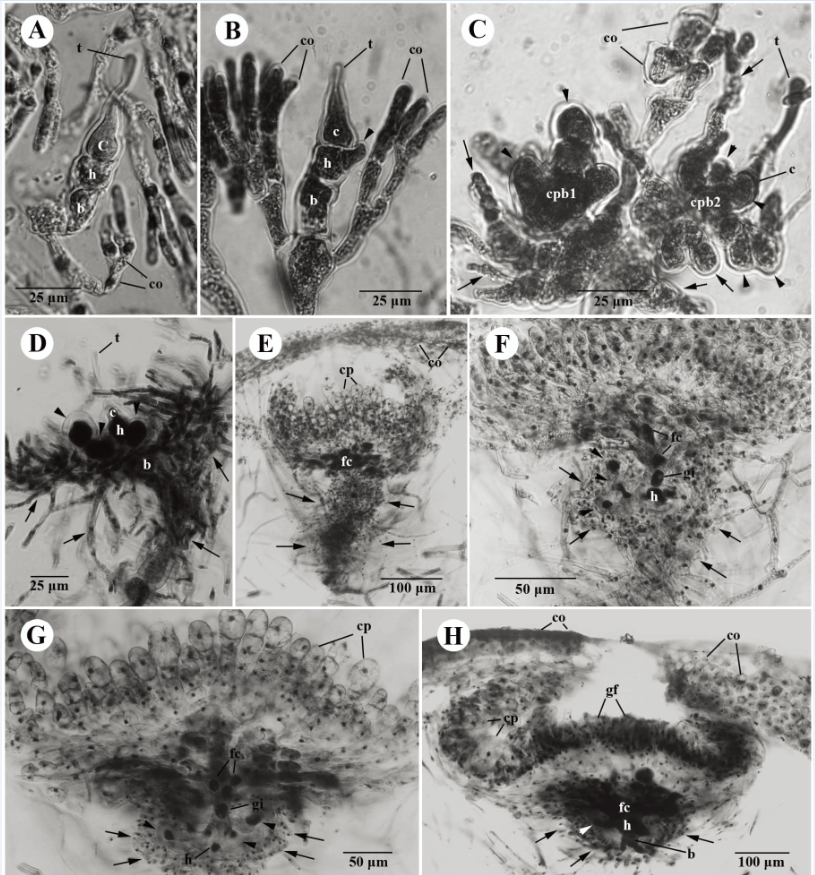

Supplement: Supplementary file 8 — Authors’ original file for figure 8 [file 40529_2011_2_MOESM8_ESM.png]

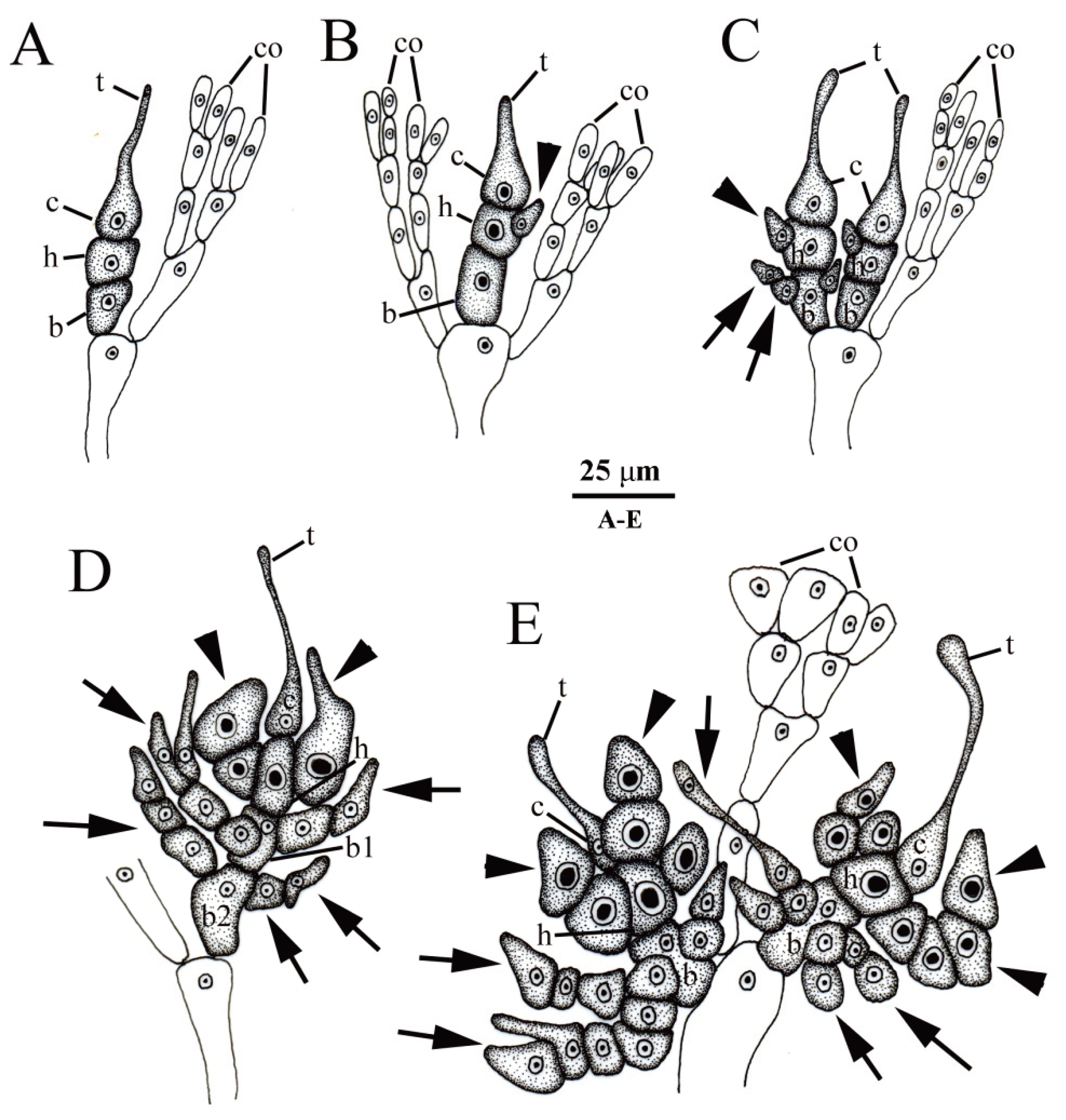

Supplement: Supplementary file 9 — Authors’ original file for figure 9 [file 40529_2011_2_MOESM9_ESM.tiff]

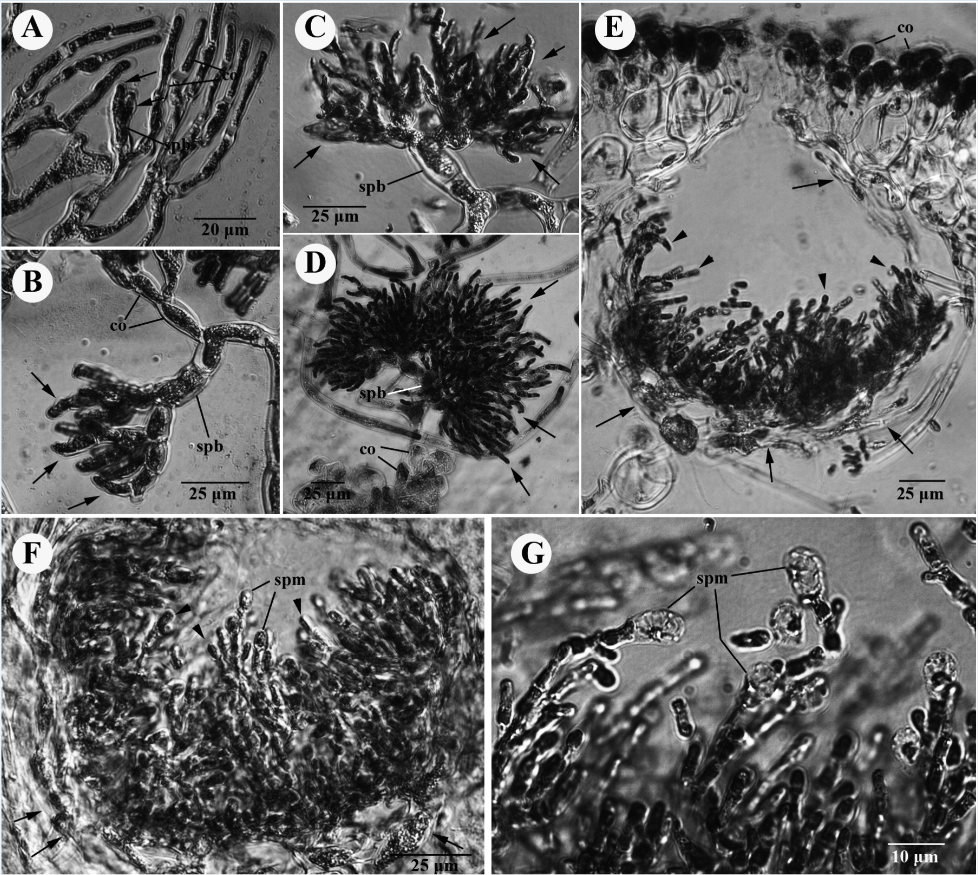

Supplement: Supplementary file 10 — Authors’ original file for figure 10 [file 40529_2011_2_MOESM10_ESM.png]
